# Switching adhesion forces by crossing the metal–insulator transition in Magnéli-type vanadium oxide crystals

**DOI:** 10.3762/bjnano.2.8

**Published:** 2011-01-27

**Authors:** Bert Stegemann, Matthias Klemm, Siegfried Horn, Mathias Woydt

**Affiliations:** 1 University of Applied Sciences (HTW) Berlin, Wilhelminenhofstr. 75a, D-12459 Berlin, Germany; 2 BAM - Federal Institute for Materials Research and Testing Berlin, Unter den Eichen 44–46, D-12203 Berlin, Germany; 3 University Augsburg, Universitätsstr. 1, D-86135 Augsburg, Germany

**Keywords:** adhesion force, atomic force microscopy, Magnéli phases, metal–insulator transition, vanadium oxide

## Abstract

Magnéli-type vanadium oxides form the homologous series V*_n_*O_2_*_n_*_-1_ and exhibit a temperature-induced, reversible metal–insulator first order phase transition (MIT). We studied the change of the adhesion force across the transition temperature between the cleavage planes of various vanadium oxide Magnéli phases (*n* = 3 … 7) and spherical titanium atomic force microscope (AFM) tips by systematic force–distance measurements with a variable-temperature AFM under ultrahigh vacuum conditions (UHV). The results show, for all investigated samples, that crossing the transition temperatures leads to a distinct change of the adhesion force. Low adhesion corresponds consistently to the metallic state. Accordingly, the ability to modify the electronic structure of the vanadium Magnéli phases while maintaining composition, stoichiometry and crystallographic integrity, allows for relating frictional and electronic material properties at the nano scale. This behavior makes the vanadium Magnéli phases interesting candidates for technology, e.g., as intelligent devices or coatings where switching of adhesion or friction is desired.

## Introduction

Thermally controlled metal–insulator transitions (MIT) are observed in a large number of crystalline and amorphous semiconductors. Particularly among the transition metal oxides, there are numerous compounds with partially filled electron bands, which show insulator behavior at low temperatures, although they should be metals with respect to the band model. Well-known examples are Magnéli-type vanadium oxide compounds, which form the homologous series V*_n_*O_2_*_n_*_-1_ (3 ≤ *n* ≤ 10) and which undergo an abrupt transition from metallic to insulating behavior and vice versa by a change of external parameters such as doping, pressure or temperature, even although the global stoichiometry remains unchanged [1–2]. Thereby, the electrical resistance changes by many orders of magnitude. The physical reason for this metal–insulator transition (MIT) is the correlation of d band electrons of opposite spins as explained by the Mott–Hubbard model [3].

It was first recognized by Magnèli et al., that oxides of titanium and vanadium as well as those of molybdenum and tungsten form homologous series with planar faults of general formulae (Ti,V)*_n_*O_2_*_n_*_-1_ or (W,Mo)*_n_*O_3_*_n_*_-1_ [4–6]. In a simplified way, the Magnèli phase structure can be derived from a perfect V_2_O_5_ crystal, which has one missing oxygen layer, i.e., the (121) plane, which is called the crystallographic shear (CS) plane and compensates for the non-stoichiometry of the compounds. The different stoichiometries result from different spacings between the CS planes and appear to be stable at high temperature before dissolving as point defects. The CS planes interact over rather large distances (≈100 Å or more) to form regular or nearly regular arrays in an otherwise perfect crystal. The overall stoichiometry of the resulting crystals depends upon the width of the particular crystallographic plane in which the CS occurs. As a consequence, a homologous series of structures is formed [7–8].

The special electrical as well as optical properties of the Magnèli phases are of great interest not only for basic research but also for future technological applications [9–11]. Therefore, materials with correlated electrons play a major role, e.g., for the construction of switches and sensors and, more generally, for the development of novel electronic devices and micro-electro-mechanical systems (MEMS). In this context, a great technological challenge in advancing miniaturization is to overcome the strong adhesive attractions between nanoscopic tribo-elements in order to realize technical systems with low friction [12–13].

The atomic force microscope (AFM) has become a powerful tool for measuring the forces interacting between a sharp tip and a solid sample surface, such as van der Waals forces and short-range chemical forces [14–17]. Typically, the AFM is used for a spatially resolved imaging of forces, which requires a tip with a sharp apex. However, such tips are disadvantageous for quantitative measurements of interfacial forces, because reliable and accurate determination of the tip geometry and also comparison with theoretical predictions are difficult. In contrast, utilizing a microsphere attached to the free end of the cantilever instead of a sharp tip provides a well-defined, theoretically controllable sphere versus flat surface geometry for the scaling of forces [18–21]. Furthermore, it allows customizing of the probe material and size. This method, also referred to as spherical-probe or colloidal-probe AFM technique, is thus better suited for quantitative and comparative adhesion force measurements [22–24]. Previously, the applicability and the sensitivity of the AFM in the spherical probe configuration (i.e., with a microsphere as a probe tip) operated under ultrahigh vacuum conditions for the quantification of adhesion forces on metal single crystals was demonstrated [25].

In our approach, adhesion forces were assessed by sensing the force interaction between the cleavage planes of four different Magnéli-type vanadium oxide single crystals (V*_n_*O_2_*_n_*_-1_, *n* = 3, 4, 6, 7) and a micro-spherical titanium AFM probe as a function of the probe/sample separation under UHV conditions, where environmental influence is eliminated and advantage of surface preparation and analysis tools can be taken. The MIT was induced by appropriate variation of temperature. In particular, we report on the change of the adhesion force when crossing the MIT temperature and correlate this behavior to the corresponding phase transition.

## Results and Discussion

**Figure 1:**
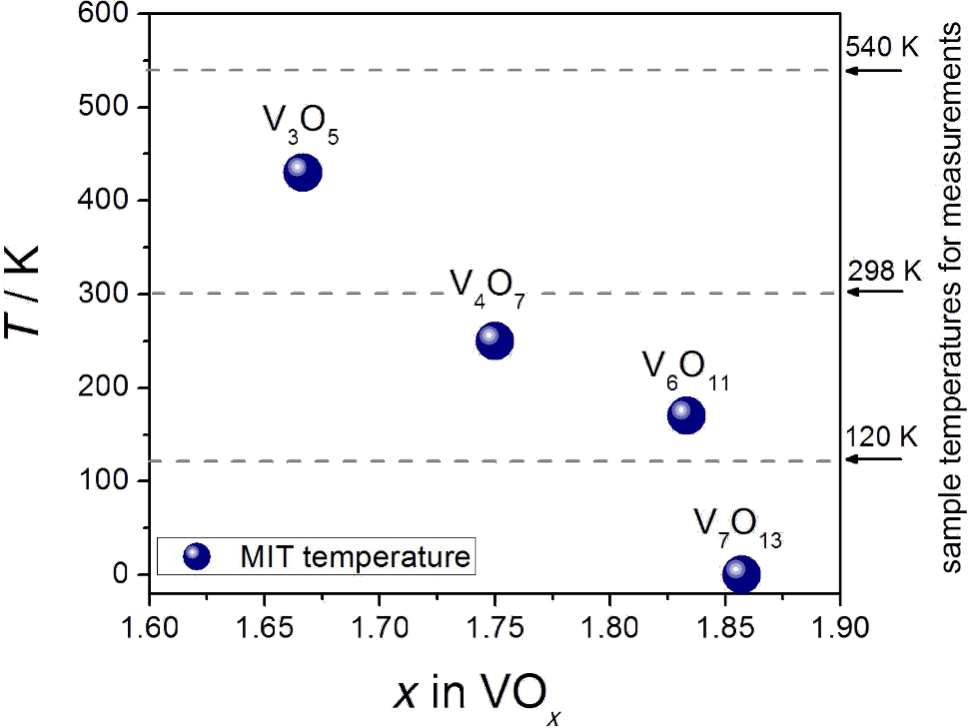
Metal–insulator transition (MIT) temperatures of the investigated Magnéli-type vanadium oxide crystals [1,26]. V_7_O_13_ does not show an MIT. The right scale shows temperatures where force measurements were carried out to probe the metallic and the insulating state of each sample.

Adhesion force measurements were carried out on the cleavage planes of the vanadium oxide both at room temperature (298 K) and at an appropriate temperature beyond the MIT. According to the measurement temperatures indicated in Figure 1, for V_4_O_7_ and V_6_O_11_ a sample temperature of 120 K and for V_3_O_5_ a sample temperature of 540 K was chosen in order to cross the MIT temperature. As a reference, V_7_O_13_ which exists solely in the metallic phase and which does not exhibit an MIT (i.e., *T*_MIT_ = 0 K) was measured at all three temperatures.

**Figure 2:**
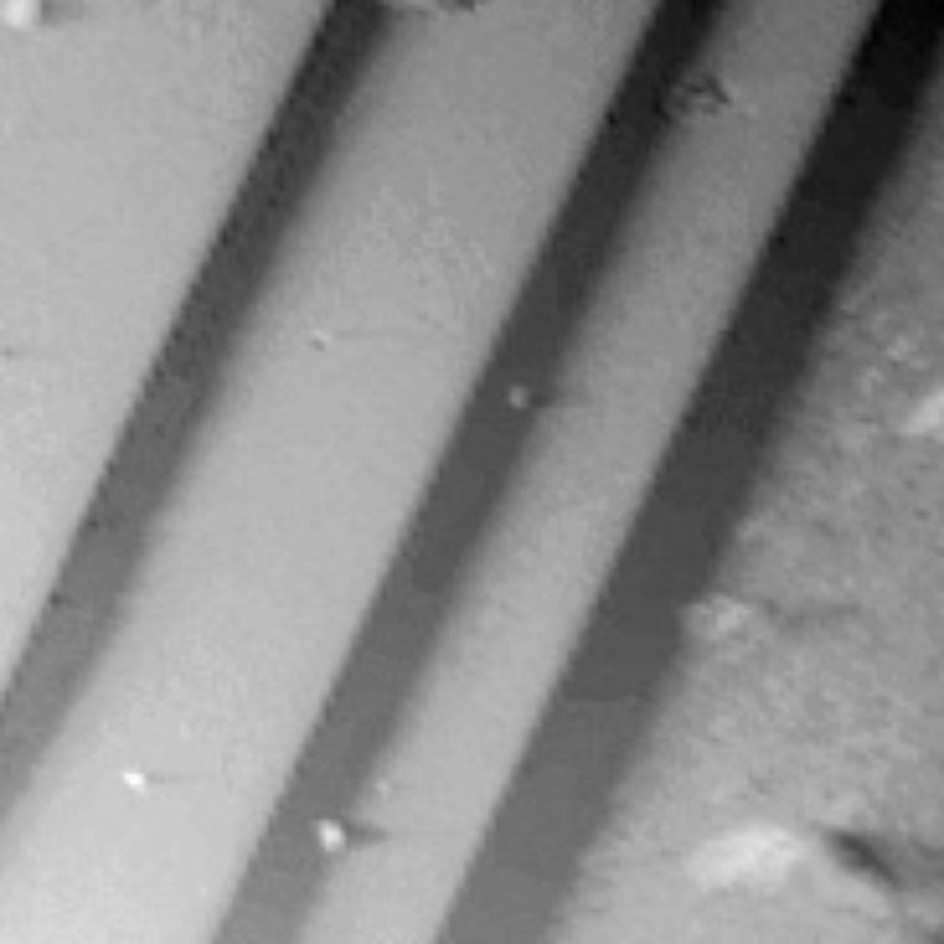
Contact mode AFM topograph of the V_4_O_7_ crystal cleavage plane. Scanning size: 25 × 25 µm^2^, *z*-range 1 µm.

Before acquisition of the force–distance curves, the topography of the vanadium oxide cleavage was characterized by contact mode AFM using a conventional sharp tip. Topography is of importance for the study of adhesion forces since all realistic surfaces normally exhibit some degree of roughness. Surface roughness is expected to decrease the actual area of contact and reduce the measured adhesion force. However, Magnéli-type vanadium oxides possess a layered structure with a planar oxygen defect [2] and can be easily cleaved to provide atomically flat substrates. This is shown in Figure 2 for the case of the V_4_O_7_ cleavage plane, exhibiting atomically flat terraces with lateral extensions of up to several microns. A rough estimation of the apparent sphere/flat surface contact area according to the Hertzian theory of deformation by taking into account the deformation properties of the materials leads to a diameter of about 40 nm [18,27]. The terraces are by far wider than this value and, thus, well suited for reliable measurements of adhesion forces.

**Figure 3:**
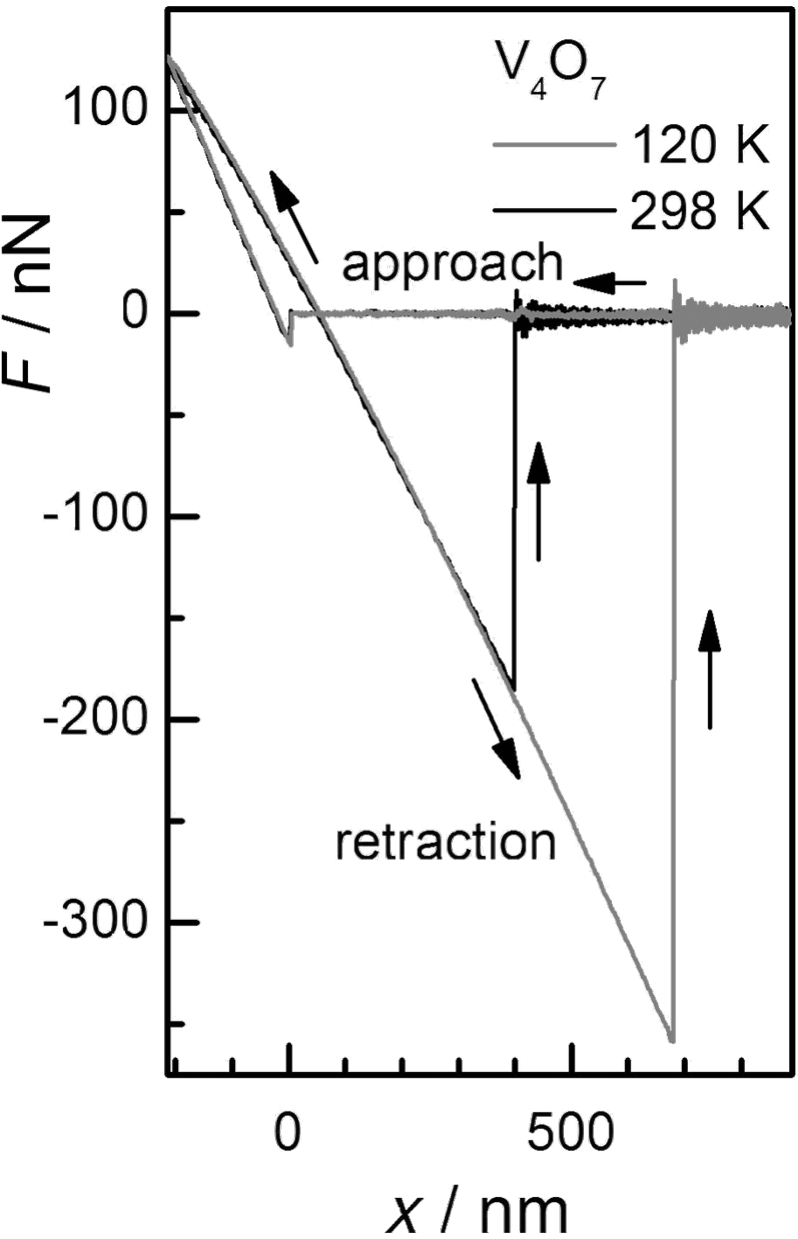
Typical force (*F*) vs distance (*x*) curves obtained on V_4_O_7_ for single measurements of a spherical Ti tip (diameter 7.2 µm) against the flat crystal plane at 120 K and 298 K. The curves show the force interaction during approach and retraction of the tip from the surface. The adhesion force corresponds to the pull-off force between the tip and sample surface.

Typical force–distance curves of single measurements obtained on V_4_O_7_ above and below the MIT temperature are shown in Figure 3. The plot shows the force interaction during approach and retraction of the spherical AFM tip from the sample surface. During retraction the tip adheres to the sample until the spring constant of the cantilever overcomes the adhesion force and the cantilever instantaneously jumps out of contact back into its equilibrium position. The force necessary to pull-off the cantilever represents, to a first approximation, the adhesion force [24,28].

**Figure 4:**
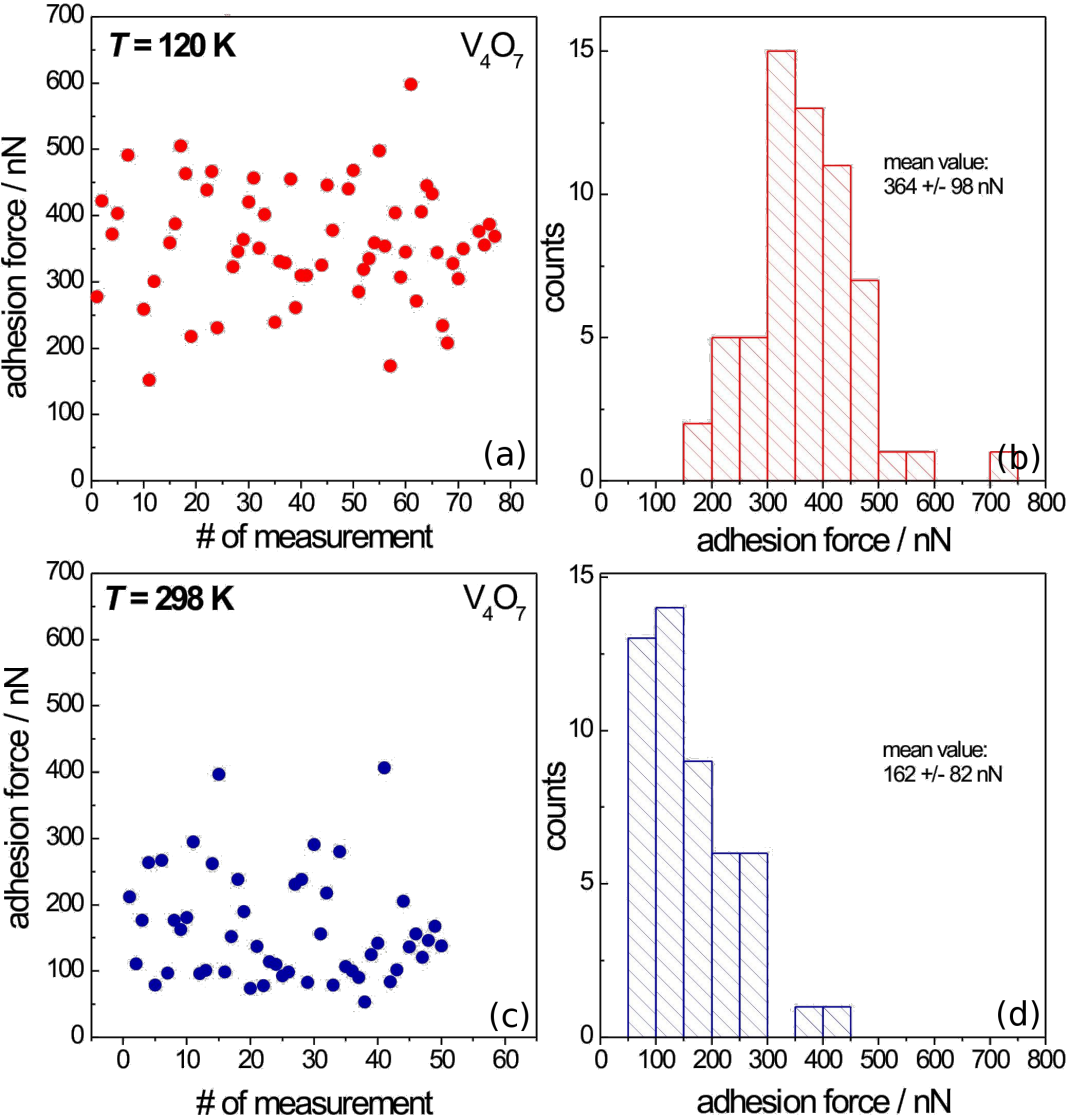
Statistical analysis of the adhesion forces acquired at the V_4_O_7_ cleavage plane at (a, b) 120 K and (c, d) 298 K. Displayed are (a, c) the sequences of data points acquired at different surface spots and (b, d) the normalized frequency distributions. The numbers given are the data averages and their standard deviations.

The graphs in Figure 4 provide an analysis of the adhesion forces acquired at the V_4_O_7_ cleavage plane at 120 K (i.e., below the MIT temperature) and at 298 K (i.e., above the MIT temperature). Displayed are the sequences of the measured data and the frequency distributions for both temperatures. Some 50 to 80 force measurements at different spots all over the surface were made. All measurements were carried out at intermediate retraction velocities and at low loads, so that the behavior of the contact is dominated by the action of surface forces [29–30]. Each data point was checked for reproducibility by at least two consecutive measurements.

**Figure 5:**
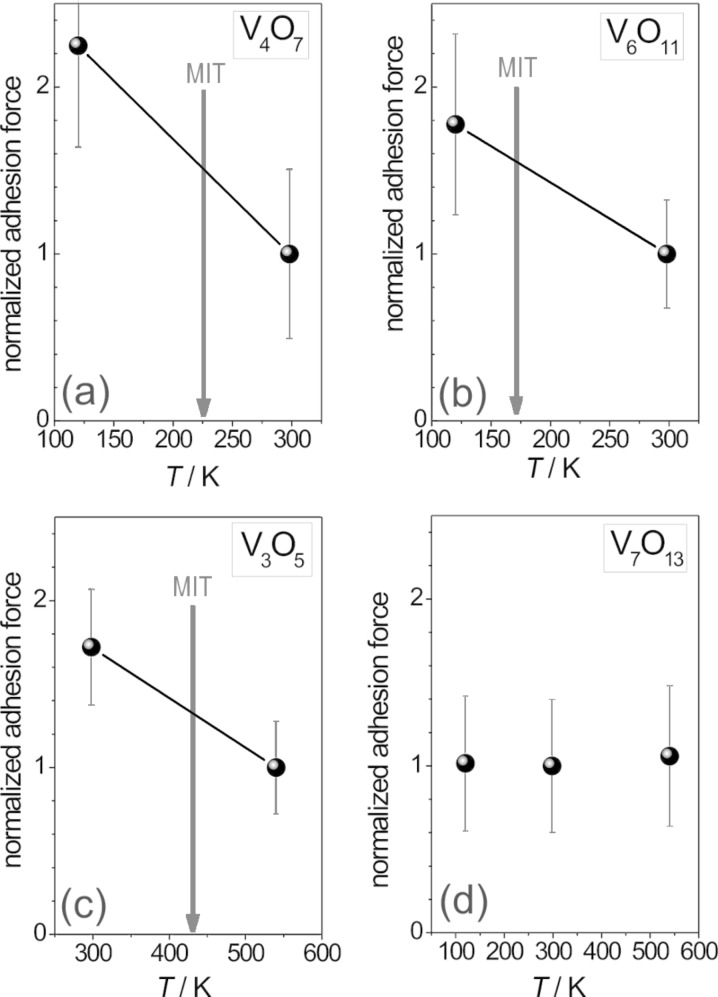
Summary of the mean values of the adhesion forces for all investigated Magnéli phases above and below the MIT temperature. Error bars represent the standard deviations. Each data point comprises reproducible measurements at 50 to 80 different spots. Values are normalized to facilitate comparison between different samples. All measurements on the same sample were performed with the same tip.

It was found that throughout the measurements on the same surface spot the adhesion force remains rather constant, indicating that the tip did not change significantly during successive force curve acquisition. However, when acquiring force–distance curves at different positions on the surface plane there was some scatter in the data. This scatter might be explained by topographic effects, i.e., interaction with cleavage steps (cf. Figure 2) or slight surface heterogeneities resulting in variations of the interaction geometry. The values given in the graphs (right column) are the data averages and their standard deviations. By comparing these two curves, it is instantly obvious that the adhesion force below the MIT is significantly higher than above the MIT (cf. Figure 5a). Accordingly, the lower adhesion force corresponds to the metallic vanadium oxide phase. Since contact models of a sphere/flat surface geometry [19–20] predict a linear dependence of the adhesion force on the sphere radius, all measured adhesion forces are normalized in this graph to the value obtained above the MIT temperature – corresponding to the metallic phase. Thus comparison between measurements carried out with different micro-spherical tips is facilitated. Error bars correspond to the standard deviation of the mean value as obtained from the statistical data analysis (cf. Figure 4).

For the measurements on the cleavage plane of the V_6_O_11_ crystal, as well as of the V_3_O_5_ and V_7_O_13_ crystals, the same procedure of data acquisition and data evaluation was followed. The summary of the statistical analysis of the adhesion forces acquired on V_6_O_11_ at 120 K and 298 K, respectively, is shown in Figure 5b. Again, there is a distinct jump in the adhesion going to low temperatures and crossing the MIT temperature. This is well in accord with the results on V_4_O_7_. According to Figure 2, for V_3_O_5_ the sample temperature had to be raised above 430 K in order to cross the MIT temperature. As shown in Figure 5c, in this case the adhesion force significantly drops, which is consistent to the observations on the previous samples because again the metallic phase exhibits the lower adhesion force. V_7_O_13_ is known to undergo no phase transition neither when the sample is cooled down nor when heated up. Rather it maintains its metallic state. Due to this feature the V_7_O_13_ phase acted as reference sample in order to prove that the observed jump in the adhesion force is not simply a temperature-related artifact but rather due to the phase transformation in the crystal. The measurements reveal that in this case the adhesion force remains indeed constant when going to high or low temperatures (see Figure 5d). This behavior clearly indicates that the jump in the adhesion force is correlated to the change in the electronic properties of vanadium oxide crystals due the phase transformation when crossing the MIT temperature.

For the investigated Magnéli-type oxides with a MIT, the adhesion force of the insulating phase is roughly twice as high as in the metallic state. This behavior is in contrast to observations at the macroscale [31], but it can be related to the distortion of the crystal structure and the distinct change of conductivity occurring at the MIT. In theory, the interaction of an ideal sphere with an atomically flat surface is, e.g., described by the Derjaguin–Muller–Toporov (DMT) model [20] or the Johnson–Kendall–Roberts (JKR) model [19]. These two models improved the Hertzian theory [18] by including the effect of adhesion and present the limiting cases of more general contact theories by Maugis [32]. Both models have in common that the pull-off-force is independent of the elastic material properties but is essentially a linear function not only of the sphere radius but also of the surface energy of the sample. At the MIT the crystal structure of the Magnéli phases is distorted resulting in a slightly higher density in the metallic phase [33] and an increase of the atomic density at the surface. A decrease of the surface energy [34] and hence a decrease of the adhesion force is expected, as was observed in the experiments.

Furthermore, the distinct increase of conductivity will lead to a better screening of trapped charge defects in the surface and therefore decrease the electrostatic contribution of the overall adhesion force. However, reference measurements with a silica microsphere on V_3_O_5_ showed the same qualitative behavior, i.e., a lower adhesion force in the metallic state. Accordingly, a possible tip-induced electrostatic contact charging is negligible.

## Conclusion

The adhesion forces of Magnéli-type phases of vanadium oxide, acquired by means of force–distance measurements with a spherical AFM probe, show a distinct response to the temperature-induced metal–insulator transition. This behavior makes the vanadium Magnéli phases interesting candidates for technological applications where switching of adhesion or friction is desired, such as intelligent devices or coatings. At the nanoscale, these adhesion measurements displayed a lower adhesion force in the metallic state than in the non-metallic, ceramic state, which is in contrast to the macroscopic experience in tribology. In accord with several recent examples, this study indicates that tribological properties at the nanoscale cannot be predicted directly from macroscopic laws [35]. Detailed measurements are in progress to obtain a better understanding of the observed phenomenon. An extension of this study to further materials revealed consistent results: Comparative adhesion force measurements of the (0001) basal planes and the (10−10) prism planes of highly oriented pyrolytic graphite (HOPG) and MoS_2_ also showed that the metallic state lowers the adhesion at the nanoscale [36].

## Experimental

### Vanadium oxide crystal preparation

Single crystals of the vanadium oxide Magnéli phases were grown in vacuum sealed quartz tubes in a gradient furnace. The chemical transport reaction, using TeCl_4_ as a transport agent took nearly six weeks. The growth temperature was 600 °C. The different phases were prepared by adjusting the oxygen content by means of a definite mixture of the starting vanadium oxides V_2_O_3_ and VO_2_ [37]. Under such conditions crystals of exclusively one Magnéli phase per tube could be obtained, several of which showed specular surfaces. X-Ray diffraction (XRD) and magnetic susceptibility measurements on representative crystals of all the batches were carried out to characterize the quality of the crystals. The MIT temperatures of the samples under study in the present work are displayed in Figure 1.

### Spherical AFM probe preparation

**Figure 6:**
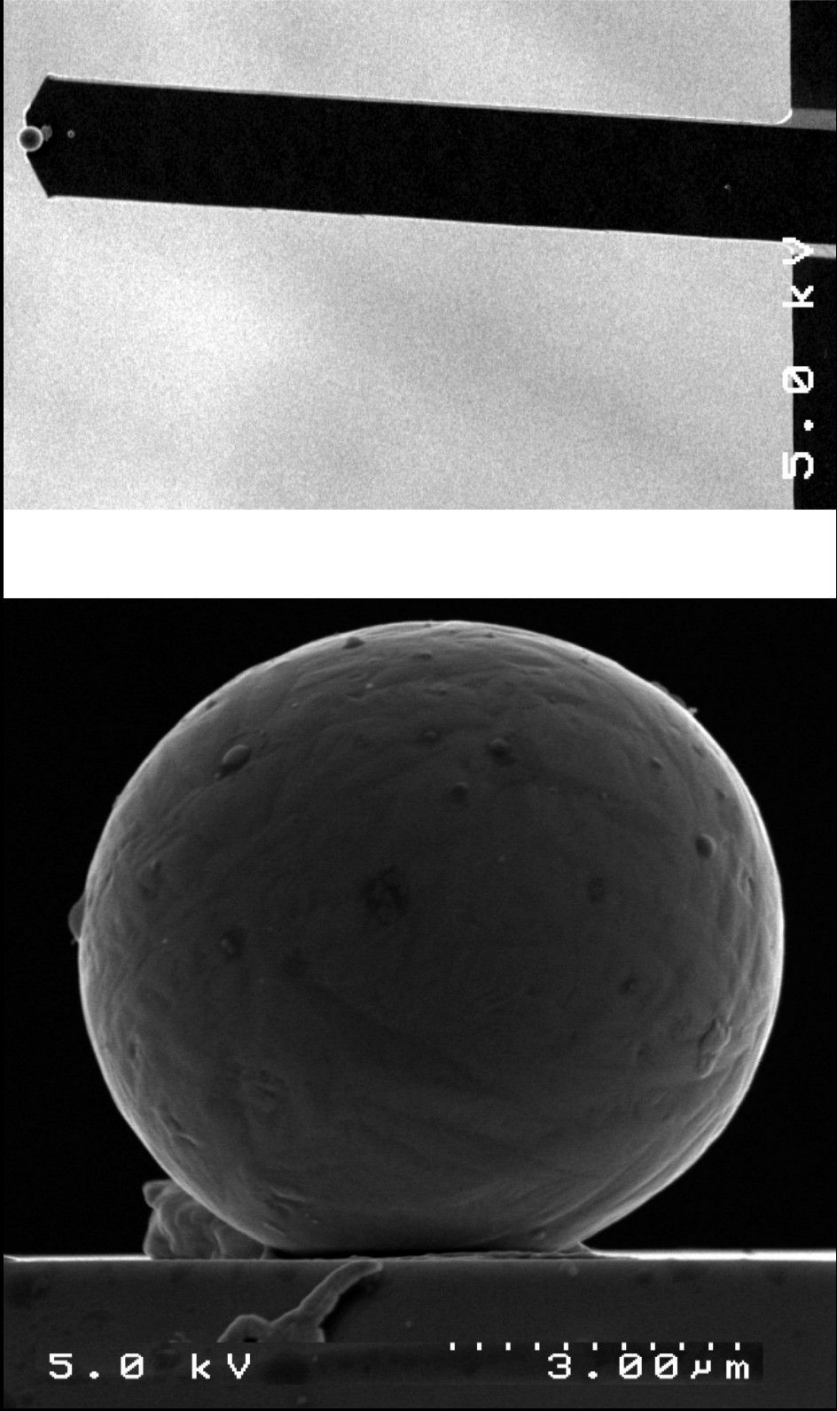
SEM images of a Ti microsphere (diameter 7.2 µm) attached at the free end of a single beam tipless AFM cantilever.

The spherical AFM probes were prepared by attaching a microsphere of the desired size and material to the end of AFM cantilevers using an *x-y-z*-micromanipulator and an optical microscope. For the experiments presented here, titanium microspheres (Alfa Aesar GmbH) were conductively glued to the free end of tipless NSC12 cantilevers (Silicon-MDT Ltd.). The successful attachment of the spheres was verified by scanning electron microscopy (SEM), as shown in Figure 6. The titanium microspheres have a smooth surface and show normally an elastic response. In situ characterization of the spherical tips was performed by reverse tip imaging with the calibration grating TGT01 (Silicon-MDT Ltd.), which consists of an array of sharp spikes [25,38–39]. Scanning this grating with a spherical AFM probe creates an image consisting of an array of spherical caps, i.e., the microsphere itself is imaged repeatedly by each spike in the scanning area. This technique allows the precise determination of the shape and radius of the microsphere. Moreover, there is the possibility of easy in situ re-examination of the spherical probe under UHV conditions to reveal shape deformation or material take-up possibly occurring during the experiment.

### Adhesion force measurements

The crystals were cleaved under ambient conditions, then immediately transferred into the UHV apparatus and degassed for a few hours. The UHV apparatus consists of two directly coupled chambers with a base pressure of <6 × 10^−11^ mbar. The preparation chamber is equipped with sample heating and cleaning faculties. The analysis chamber houses a variable temperature scanning probe microscope (Omicron Nanotechnology, Germany), which allows AFM measurements at sample temperatures in the range from 120 K to 1000 K by either cooling with liquid N_2_ or radiative heating. Temperature measurements were made with a thermocouple attached to the sample acceptance stage. The actual temperature of the sample plates is taken from a calibration curve with an accuracy of ±20 K as provided by the manufacturer.

The spring constant of the cantilevers with attached microsphere (typically 3.0 ± 0.2 N/m) was determined by means of the reference cantilever technique, where the cantilever under test was deflected in situ against a cantilever with a precisely known spring constant [40–41]. The spring constant of the reference cantilever (Park Scientific Instruments) was determined by a calculation based on geometrical dimensions and resonance frequency, as determined from SEM images and scanning laser vibrometry measurements, respectively.

For reliable comparison of the data acquired on a certain sample, only adhesion forces obtained with one and the same tip were taken into account. To facilitate comparison between different samples where different spherical tips had to be used, adhesion force values were normalized.
